# Longitudinal association between CRP levels and risk of psychosis: a meta-analysis of population-based cohort studies

**DOI:** 10.1038/s41537-021-00161-4

**Published:** 2021-05-28

**Authors:** Emanuele F. Osimo, Luke Baxter, Jan Stochl, Benjamin I. Perry, Stephen A. Metcalf, Setor K. Kunutsor, Jari A. Laukkanen, Marie Kim Wium-Andersen, Peter B. Jones, Golam M. Khandaker

**Affiliations:** 1 Department of Psychiatry, University of Cambridge, Cambridge, UK; 2 MRC London Institute of Medical Sciences, Institute of Clinical Sciences, Imperial College London, London, UK; 3 Cambridgeshire and Peterborough NHS Foundation Trust, Cambridge, UK; 4 Barking, Havering and Redbridge University Hospitals NHS Trust, Romford, UK; 5 Department of Kinanthropology and Humanities, Charles University, Prague, Czech Republic; 6 Department of Public Health and Primary Care, University of Cambridge, Cambridge, UK; 7 National Institute for Health Research Bristol Biomedical Research Centre, University Hospitals Bristol and Weston NHS Foundation Trust and the University of Bristol, Bristol, UK; 8 Translational Health Sciences, Bristol Medical School, University of Bristol, Learning & Research Building (Level 1), Southmead Hospital, Bristol, UK; 9 Institute of Public Health and Clinical Nutrition, University of Eastern Finland, Kuopio, Finland; 10 Institute of Clinical Medicine, Department of Medicine, University of Eastern Finland, Kuopio, Finland; 11 Central Finland Health Care District, Department of Medicine, Jyväskylä, Finland; 12 Center for Clinical Research and Prevention, Bispebjerg and Frederiksberg Hospital, Frederiksberg, Denmark; 13 Department of Clinical Biochemistry, Herlev and Gentofte Hospitals, Herlev, Denmark; 14 Psychiatric Center Ballerup, Ballerup, Denmark; 15 Applied Research Collaboration East of England, National Institute for Health Research (NIHR), England, UK; 16 MRC Integrative Epidemiology Unit, Population Health Sciences, Bristol Medical School, University of Bristol, Bristol, UK; 17 Centre for Academic Mental Health, Population Health Sciences, Bristol Medical School, University of Bristol, Bristol, UK

**Keywords:** Biomarkers, Psychosis

## Abstract

Meta-analyses of cross-sectional studies suggest that patients with psychosis have higher circulating levels of C-reactive protein (CRP) compared with healthy controls; however, cause and effect is unclear. We examined the prospective association between CRP levels and subsequent risk of developing a psychotic disorder by conducting a systematic review and meta-analysis of population-based cohort studies. Databases were searched for prospective studies of CRP and psychosis. We obtained unpublished results, including adjustment for age, sex, body mass index, smoking, alcohol use, and socioeconomic status and suspected infection (CRP > 10 mg/L). Based on random effect meta-analysis of 89,792 participants (494 incident cases of psychosis at follow-up), the pooled odds ratio (OR) for psychosis for participants with high (>3 mg/L), as compared to low (≤3 mg/L) CRP levels at baseline was 1.50 (95% confidence interval [CI], 1.09–2.07). Evidence for this association remained after adjusting for potential confounders (adjusted OR [aOR] = 1.31; 95% CI, 1.03–1.66). After excluding participants with suspected infection, the OR for psychosis was 1.36 (95% CI, 1.06–1.74), but the association attenuated after controlling for confounders (aOR = 1.23; 95% CI, 0.95–1.60). Using CRP as a continuous variable, the pooled OR for psychosis per standard deviation increase in log(CRP) was 1.11 (95% CI, 0.93–1.34), and this association further attenuated after controlling for confounders (aOR = 1.07; 95% CI, 0.90–1.27) and excluding participants with suspected infection (aOR = 1.07; 95% CI, 0.92–1.24). There was no association using CRP as a categorical variable (low, medium or high). While we provide some evidence of a longitudinal association between high CRP (>3 mg/L) and psychosis, larger studies are required to enable definitive conclusions.

## Introduction

Several lines of evidence implicate infection and inflammation in the pathogenesis of schizophrenia and related psychotic disorders. First, childhood infections involving the central nervous system^1,2^, and particularly viral infections^1,3^, are associated with a nearly twofold risk of adult schizophrenia^4^. Second, meta-analyses of population-based longitudinal studies suggest that prenatal maternal infection, including bacterial^5^, respiratory^6^, or genital and reproductive infections^5,7,8^, are associated with a two- to fivefold increased risk of schizophrenia in the offspring. Third, meta-analyses of cross-sectional studies have reported raised levels of several inflammatory markers and elevated specific cell counts in patients with schizophrenia as compared to controls^9–16^.

C-reactive protein (CRP) is an acute-phase protein and an archetypal inflammatory marker that has been used most extensively as a proxy for systemic inflammation in studies of physical^17,18^ and psychiatric disorders^14,19–25^. Meta-analyses of cross-sectional studies reported that circulating CRP levels are higher in schizophrenia compared to healthy controls, with medium to large effect sizes^13,14,26^. Our recent meta-analysis^15^ supports small to medium effect sizes in medication-naïve first-episode psychosis patients. However, cross-sectional studies cannot ascertain the direction of the association, i.e., whether elevated CRP predates or follows psychosis.

Longitudinal studies can disentangle the directionality of the association between exposure and outcome, and therefore can help to address the issue of reverse causation^27^. Furthermore, population-based longitudinal studies are less prone to sampling bias, a common issue for cross-sectional designs, which often include hospitalized patients^27^. A number of population-based studies have reported a longitudinal association between higher CRP levels at baseline and risk of psychosis at follow-up^28–34^. However, there are important issues that need to be considered to allow more definitive conclusions. For example, there is heterogeneity in sample age with some studies conducted in relatively young^29,31–34^ and some in relatively older populations^28,30^. While these studies have controlled for various confounders, variables included varied from study to study. Third, not all studies excluded participants with suspected infection, as measured by a fixed CRP threshold^28,30,33^. Therefore, a more streamlined analysis across cohorts with consistent adjustment for key confounders is required.

In this study, we aimed to examine the longitudinal associations between circulating CRP levels and subsequent risk of psychotic disorders by carrying out a systematic review and meta-analysis of population-based prospective studies. We examined the nature of this longitudinal association in a number of ways. First, we used CRP both as a continuous and a binary variable. For the latter, we defined CRP levels >3 mg/L as “high” in line with the American Heart Association and the US Centers for Disease Control and Prevention criteria for CRP use in population-based research^35^, and in line with previous psychiatric research^20–22^. Second, to examine the potential effect of confounding, we used results from each cohort before and after adjusting for the same set of confounders including age, sex, body mass index (BMI), measures of socioeconomic status (SES), smoking, and alcohol use. We repeated the analysis after excluding participants with baseline CRP levels >10 mg/L to minimise potential confounding by acute inflammation/infection^35^. Finally, to examine the effect of age we carried out sub-group analyses by age.

## Results

The literature search yielded 303 studies, out of which five met the inclusion criteria^28–31,34^. Two of these studies examined the associations of childhood CRP levels with the risk of psychotic disorder in adulthood in the ALSPAC birth cohort^29,34^. Of the two, we included the study with the longer follow-up^34^. Consequently, four studies were included in meta-analysis^28,30,31,34^. Please see Supplementary Fig. 1 for the PRISMA diagram of study selection.

### Description of cohort samples

The studies included in the meta-analysis comprised 89,792 participants, with 494 incident cases of psychosis, drawn from separate areas of Europe. Please see Table 1 for study characteristics. The studies had an average follow-up length of 14.55 years (SD = 7.58) for psychosis after CRP assessment at baseline. Two studies involved relatively young adults with psychosis from specific birth cohorts (mean age at outcome assessment around 24–26 years)^31,34^, while two involved older adults (mean age at the outcome of 64–78 years)^28,30^. Samples were, on average, 60.2% male (range: 44.5–100%). The mean percentage of white ethnicity across studies was 99.7% (range: 98.7–100%).

**Table 1:** Characteristics of Included General Population-based Prospective Studies of CRP and Psychosis.

Study	Country	Cohort	Mean (SD) age at baseline (at blood sampling), yrs	Mean (SD) age at follow-up (at psychosis diagnosis), yrs	Mean (SD) time to follow-up, yrs	Male sex (%)	White ethnicity (%)	Method of case ascertainment	Definition of outcome	Total analytic sample (N)	N of Incident cases of psychosis at follow-up (N of cases with CRP > 3 mg/L)	Analytic sample excluding CRP > 10 mg/L (N)	Incident cases of psychosis at follow-up excluding CRP > 10 mg/L (N)
Wium-Andersen et al.^28^	Denmark	Copenhagen City Heart Study and Copenhagen General Population Study	57 (13.7)	64.3 (14.0)	5.25 (4.10)	44.5	100	Hospital-based diagnoses from the national Danish Patient and the Causes of Death Registry and out-patient and emergency contacts.	Psychotic disorders according to ICD-8 (295.0-9, 296.89, 297, 298.39, and 301.83) or ICD-10 (F20.0-9, F21-F29).	78,769	131 (49)	75,989	116
Metcalf et al.^31^	Finland	Northern Finland Birth Cohort 1986	16.00 (0.38)	26.92 (0.87)	10.92 (0.85)	49.9	100^a^	Healthcare-based diagnoses from the Finnish Hospital Discharge Register and healthcare outpatient and hospital outpatient records. Inpatient and community	Non-affective psychotic disorders according to ICD-10 (F20–F29)	6,362	88 (9)	6,258	83
Laukkanen et al.^30^	Finland	Kuopio Ischaemic Heart Disease cohort	53.1 (5.1)	78 (5.1)	21.5 (7.6)	100	100	An independent committee of researchers reviewed all potential cases and assigned diagnoses. Inpatient and community	Psychotic disorders according to ICD-9 (290–299) and ICD-10 (F00–F09 and F20–F29).	2,552	245 (54)	2,463	238
Perry et al.^34^	England	ALSPAC birth cohort	9.9 (0.32)	24.04 (0.85)	15 (not available)	46.2	98.7	Face-to-face semi-structured Psychosis-Like Symptom Interview (PLIKSi) of entire cohort attending clinical assessment	Psychotic disorder^b^	2,224	30 (2)	2,203	30

^a^To the Northern Finland Birth Cohort (NFBC) 1986 research team’s knowledge, participants are mostly, if not all, of Finnish origin and white ethnicity. However, there has been no official documentation of race or ethnicity in the cohort, so 100% white ethnicity and Finnish origin is an assumption (Minna Ruddock, PhD, NFBC1986 research director, email communication, 13 July 2020).

^b^In Perry et al., cases of psychotic disorder were defined as having interviewer-rated definite psychotic episodes (PEs) that were not attributable to the effects of sleep/fever, had occurred regularly at least once per month over the previous 6 months, and were either (i) very distressing, (ii) negatively impacted social/occupational functioning, or (iii) led to help-seeking from a professional source. PEs were identified through the face-to-face, semi-structured Psychosis-Like Symptom Interview (PLIKSi) conducted by trained psychology graduates and were coded according to the definitions in the Schedules for Clinical Assessment in Neuropsychiatry, Version 2.0. PEs, occurring in the last 6 months, covering the three main domains of positive psychotic symptoms were elicited: hallucinations, delusions, and thought interference (as per Sullivan et al.^65^).

In the study by Perry et al.^34^, CRP was measured at age 9, and psychosis was assessed at age 24 using a semi-structured face-to-face interview (see Table 1 for details). Metcalf et al.^31^ used the NFBC 1986 cohort, in which CRP was measured at age 16, and psychosis were assessed up to age 27 using hospital inpatient, hospital outpatient, and healthcare outpatient records. The study by Wium-Andersen et al.^28^ included all register-based diagnoses of all hospital contacts with schizophrenia or a psychotic disorder from the Danish National Patient Registry, which includes in-patient diagnoses since 1977. In addition, psychiatric out-patient and emergency contacts since 1995 were also used to identify potential cases. In this study CRP was measured on average at age 57, and incident psychosis was detected on average up to age 64. The study by Laukkanen et al.^30^ included CRP measurements at a mean age of 53 years and data on hospitalization due to a psychotic disorder up to a mean age of 78 years, ascertained by linkage to the National Hospital Discharge Register. Diagnoses of psychotic disorders were made by qualified psychiatrists according to ICD-8, -9, and -10 codes.

Laukkanen et al. and Wium-Andersen et al.^28^ both excluded participants with psychosis at baseline. However, studies by Perry et al.^34^ and Metcalf et al.^31^ did not exclude participants with psychosis before CRP assessment at ages 9 and 16 years, respectively.

### Odds ratios (ORs) for psychosis at follow-up for individuals with high baseline CRP (>3 mg/L)

**Fig. 1:**
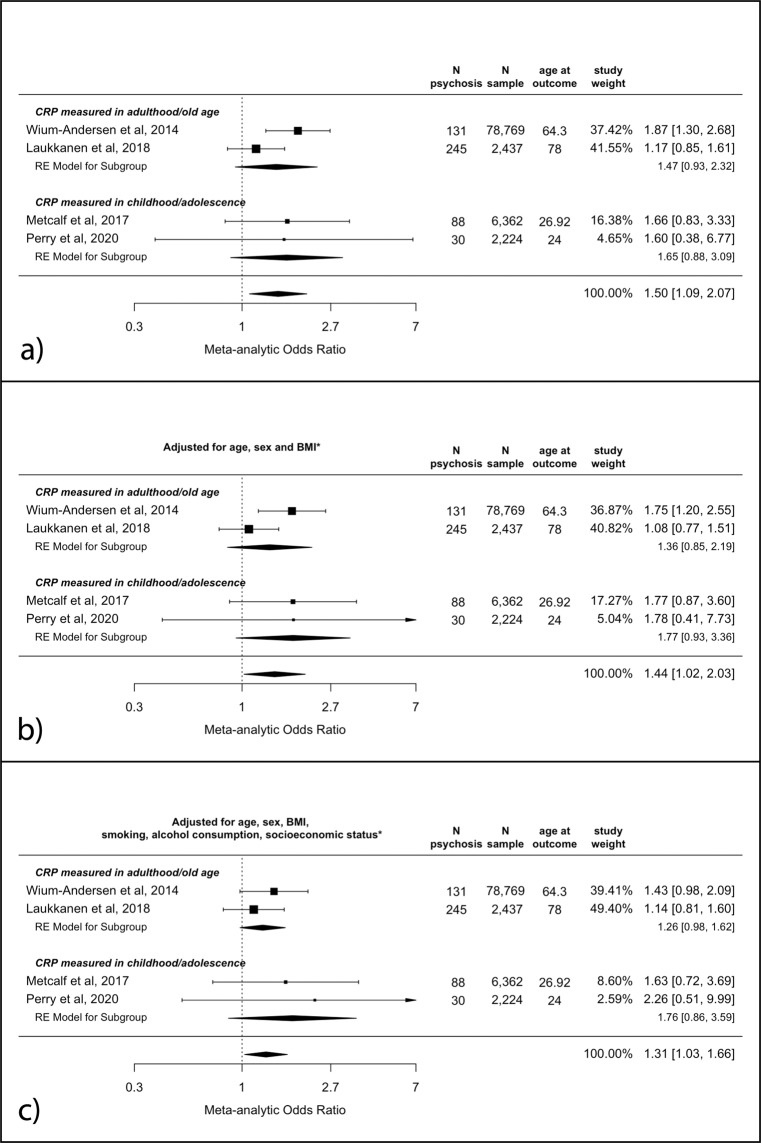
Odds ratios for psychosis at follow-up for individuals with high (>3 mg/L), as compared to low (≤3 mg/L), CRP levels at baseline. **a** Unadjusted analysis; **b** adjusted for age, sex and BMI; **c** adjusted for age, sex, BMI, smoking, alcohol consumption and socioeconomic status. * see “Methods” for specific covariates used for each study. BMI body mass index, CRP C-reactive protein, mg/L milligrams per litre.

The meta-analytic unadjusted OR for psychosis at follow-up in participants with high CRP (>3 mg/L) as compared to low CRP (≤3 mg/L) at baseline was 1.50 (95% CI, 1.09–2.07, *p* = 0.01). Point estimates were slightly larger for the childhood/adolescence samples; see Fig. 1a. Evidence for this association remained after adjusting for age, sex and BMI (adjusted pooled OR [aOR] = 1.44; 95% CI, 1.02–2.03; *p* = 0.04), and after additional adjustments for alcohol consumption, smoking levels and SES (aOR = 1.31; 95% CI, 1.03–1.66; *p* = 0.03); see Fig. 1b, c.

There was no evidence of significant heterogeneity in any of the analyses (all *I*^2^ between 0 and 39.8%; Cochran’s *Q*s between 1.6 and 4.1 (df = 3); all *p*s >0.25).

### ORs for psychosis at follow-up per SD increase in baseline CRP

**Fig. 2:**
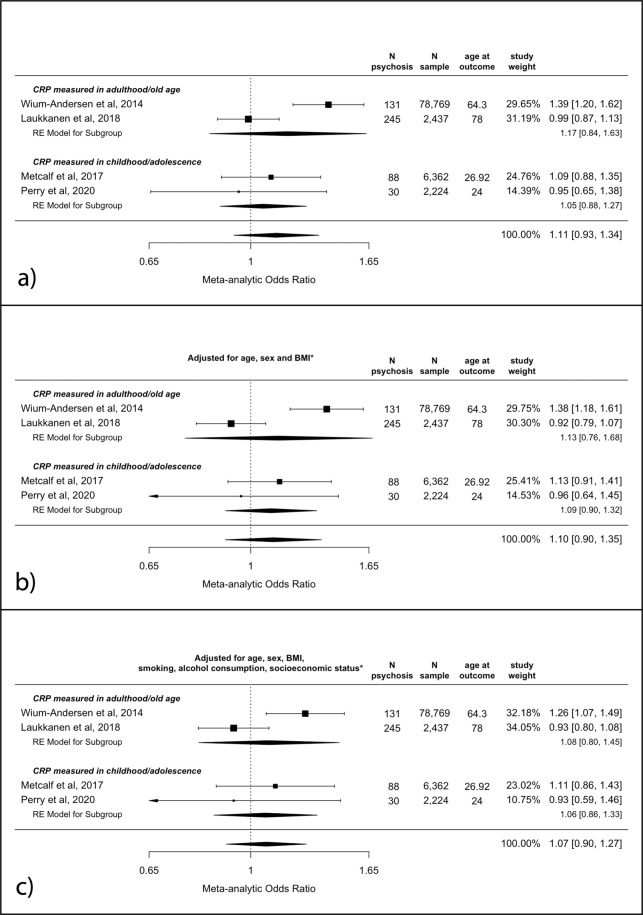
Odds ratios for psychosis at follow-up per SD increase in CRP levels at baseline. **a** Unadjusted analysis; **b** adjusted for age, sex and BMI; **c** adjusted for age, sex, BMI, smoking, alcohol consumption and socioeconomic status. * see “Methods” for specific covariates used for each study. BMI body mass index, CRP C-reactive protein, SD standard deviation.

The meta-analytic unadjusted OR for psychosis at follow-up per SD increase in baseline CRP as a continuous value was 1.11 (95% CI, 0.93–1.34, *p* = 0.24). See Fig. 2a. The 95% confidence interval widened after adjusting for age, sex and BMI (aOR = 1.10 (95% CI 0.90–1.35, *p* = 0.36). The fully adjusted model showed a pooled OR of 1.07 (95% CI, 0.90–1.27, *p* = 0.45); see Fig. 2b, c.

There was no evidence of significant heterogeneity in any of the analyses (all *I*^2^ between 31 and 58%; Cochran’s *Q*s between 3.7 and 5.7 (df = 3); all *p*s >0.05).

### ORs for psychosis at follow-up for individuals with high (>3 mg/L) and medium (1–3 mg/L), as compared to low (<1 mg/L), CRP levels at baseline

Compared to those with low baseline CRP (<1 mg/L), the OR for psychosis at follow-up was 0.93 (95% CI, 0.68–1.27) for medium (1–3 mg/L) and 1.49 (95% CI, 0.95–2.32) for high (>3 mg/L) CRP. The overall pooled OR for psychosis for CRP ≥1 mg/L as compared to <1 mg/L was 1.18 (95% CI, 0.88–1.59, *p* = 0.26).

After adjusting for all potential confounders, the aOR for the medium CRP group was 0.90 (95% CI, 0.60–1.35) and that for the high CRP group was 1.27 (95% CI, 0.84–1.94). The overall aOR for CRP ≥1 mg/L as compared to <1 mg/L was 1.07 (95% CI, 0.79–1.43, *p* = 0.67). See Supplementary Fig. 2.

### Sensitivity analyses: ORs for psychosis at follow-up for individuals with high baseline CRP (>3 mg/L), excluding subjects with suspected infection (CRP > 10 mg/L) at baseline

After excluding subjects with suspected infection (CRP > 10 mg/L) at baseline, the meta-analytic unadjusted OR for psychosis at follow-up in participants with high CRP (>3 mg/L) as compared to low CRP (≤3 mg/L) at baseline was 1.36 (95% CI, 1.06–1.74; *p* = 0.01); see Supplementary Fig. 3, panel A. Evidence for this association weakened after adjusting for age, sex and BMI (adjusted pooled OR = 1.27; 95% CI, 0.99–1.64; *p* = 0.06) and on further adjustments for alcohol consumption, smoking levels and SES (adjusted pooled OR = 1.23; 95% CI, 0.95–1.60; *p* = 0.12); see Supplementary Fig. 3, panels B and C.

### Sensitivity analyses: ORs for psychosis at follow-up per SD increase in baseline CRP as a continuous variable, excluding subjects with suspected infection (CRP > 10 mg/L) at baseline

After excluding subjects with suspected infection (CRP > 10 mg/L) at baseline, the meta-analytic unadjusted OR for psychosis at follow-up per SD increase in baseline CRP was 1.07 (95% CI 0.92–1.24, *p* = 0.40); see Supplementary Fig. 4, panel A. The 95% confidence interval widened after adjusting for age, sex and BMI (aOR = 1.04; 95% CI, 0.88–1.24; *p* = 0.64). The fully adjusted model showed a pooled OR of 1.00 (95% CI 0.88–1.13, *p* = 0.97); see Supplementary Fig. 4, panels B and C.

### Sensitivity analyses: ORs for psychosis at follow-up for individuals with high (>3 mg/L) and medium (1–3 mg/L), as compared to low (≤1 mg/L), CRP levels at baseline, excluding subjects with suspected infection (CRP > 10 mg/L) at baseline

After excluding subjects with suspected infection (CRP > 10 mg/L) at baseline, the results were similar to the primary analyses, indicating no association between high or medium CRP levels and psychosis risk (Supplementary Fig. 5).

### Sensitivity analyses: study quality assessment

All included studies were rated ≥7 on the Newcastle-Ottawa Scale for study quality (ref. ^36^; see Supplementary Table 1). After converting the scores into Agency for Healthcare Research and Quality standards (good, fair, poor)^15^, all studies were rated as ‘good quality’.

## Discussion

In this study, we meta-analyzed data from four population-based prospective studies comprising 89,792 participants and 494 incident cases of psychosis to examine the longitudinal associations between CRP at baseline and risk of psychosis at follow-up. Our results suggest that high CRP levels at baseline (>3 mg/L) are associated with an increased risk of psychosis at follow-up. However, we found no association between CRP and psychosis using CRP as a continuous variable (per SD increase), or using CRP as a categorical variable (low, medium or high). Overall, the effect sizes were slightly larger for younger compared with older samples, but our analysis included a limited number of studies and a relatively small number of cases. Therefore, in the future larger samples will be required before definitive conclusions can be drawn about the true existence of longitudinal associations between CRP and psychosis risk, and the potential effects of age of onset.

Using CRP as a binary variable, we report an increase of 50% in the odds of developing psychosis for those with high (>3 mg/L), as compared to low (≤3 mg/L), CRP levels at baseline in unadjusted analyses. The availability of unpublished results allowed us to examine the effects of a number of important confounding factors, including suspected infection (CRP > 10 mg/L) at baseline (time of blood sampling). In addition to sex, age and BMI, we included relevant social and lifestyle factors, including alcohol consumption, smoking, and SES. Our results suggest that evidence for an association between high, as compared to low, CRP levels at baseline and risk of psychosis at follow-up remained after adjusting for potential confounders. After excluding participants with suspected infection, defined as having CRP > 10 mg/L at baseline, the evidence for an association remained only in the unadjusted, but not in the adjusted, analyses.

Our findings, that dichotomous CRP levels (>3 mg/L) could be longitudinally associated with a higher risk of psychosis, are consistent with evidence from previous cross-sectional studies. These have found elevated CRP levels in first-episode psychosis as compared to healthy controls^14,37^, and in patients with chronic schizophrenia^13,38^. CRP levels have also been reported to correlate with psychotic symptom severity^14^. Potential explanations for the null findings in sensitivity analyses excluding subjects with CRP > 10 mg/L at baseline, and using CRP as a categorical variable (low, medium and high), may include limited statistical power due to a smaller number of cases overall, or in each category. We also observe no associations using CRP as a continuous variable. It is possible that the CRP-psychosis association is non-linear, as we observe an association using CRP as a binary variable but not using CRP as a continuous variable. In future, further studies with a larger number of cases will be required to test whether an association exists, and, if so if it is linear or non-linear.

A number of meta-analyses have reported that patients with schizophrenia and related psychotic disorders have higher CRP levels compared to controls^13,14^. Our findings are consistent with these results. We provide some evidence for a longitudinal association between high CRP levels at baseline and increased risk of psychosis at follow-up. However, this epidemiological evidence is at odds with recent findings from Mendelian randomization (MR) studies that used genetic variants as proxies for CRP to examine whether the CRP-psychosis association is likely to be causal or could be attributable to confounding. Using genetic variants associated with CRP levels, MR studies have reported that genetically predicted higher CRP levels are associated with a decreased risk of psychosis^39–41^. This is puzzling, given that case-control studies have consistently reported higher CRP levels in psychosis patients, and further research is required to understand the exact mechanisms.

There may be a number of potential explanations for the divergent findings between epidemiological and MR studies. For instance, it has been proposed that a genetic predisposition for a decreased acute phase response (as reflected by lower CRP levels) might increase the risk of psychosis by increasing susceptibility to infection^40,42^. The MR results could also be a consequence of not taking into account other inflammatory markers that influence CRP levels. For instance, a recent MR study by Perry and colleagues reported that, after controlling for genetically predicted levels of IL-6, IL-6 remained associated with schizophrenia while the apparent protective effect of CRP on schizophrenia risk completely attenuated^43^. This highlights the importance of considering the immune system as a highly interlinked and complex entity rather than looking at individual biomarkers in isolation.

Evidence supporting a role for inflammation in psychosis may inform novel approaches to the treatment and prevention of this condition. A meta-analysis of clinical trials of anti-inflammatory drugs given as an adjunct to antipsychotics shows that the addition of anti-inflammatories results in greater improvements in positive and negative symptoms of psychosis, as compared to antipsychotic monotherapy^44^. Further research is needed to test whether treating inflammation has broader therapeutic benefits in psychosis, and if the benefits might extend to all or just to a subgroup of patients with psychosis.

Among the strengths of this work, all the included studies were rated as “good quality” according to Agency for Healthcare Research and Quality standards. There was little evidence of heterogeneity in the meta-analyses. We used harmonized, unpublished results from each cohort to specifically address confounding, and we were able to run sensitivity analyses excluding subjects with suspected infection. We examined associations separately in cohorts including early- and late-onset psychosis. Our choice of potential confounders was informed by previous research. Both age^45–47^ and sex^47,48^ are separately associated with schizophrenia risk and levels of CRP. A higher BMI has been associated with increased CRP levels^49,50^, and BMI shows differential associations with psychosis risk depending on age. A low BMI in childhood is associated with an increased risk of psychosis^51^. Patients with psychosis tend to have higher BMI as a group possibly due to lifestyle and other factors, including metabolic effects of certain antipsychotic drugs^52^. BMI levels have also been associated with differing psychiatric outcomes in first-episode psychosis^53^, though this association was not replicated^54^. In addition, we included smoking and alcohol use as potential confounders, both of which are known to increase levels of inflammatory markers and are also associated with psychosis risk^55^. Finally, SES is an important correlate of psychosis risk^47^.

Limitations of the work include the small number of studies available to include in the meta-analysis. Therefore, formal assessment of publication bias was not appropriate, as Cochrane recommends that “tests for funnel plot asymmetry should be used only when there are at least 10 studies included in a meta-analysis”^56^.

Further, it is possible that studies with null findings were not published. The limited number of studies also precluded the use of meta-regression to explore further potentially confounding effects such as age at onset, which was instead represented as an exploratory subgroup analysis.

Our sample included a total of 494 incident cases of psychosis, of which 114 had high CRP levels at baseline (>3 mg/L), which represents 23% of the psychosis sample. This is very close to the meta-analytic estimate of the prevalence of high CRP in psychosis (28% in ref. ^13^, a meta-analysis including both inpatient and community samples), and in depression (24% in ref. ^20^, in a sensitivity analysis of community samples only).

Most study participants were of white ethnicity, which favours comparability of the included studies, but makes it less generalizable to other ethnicities, especially given the significant role of non-white ethnicity both on the risk of psychosis^47^ and on CRP levels^57^.

Furthermore, the studies included substantial levels of heterogeneity with regard to the estimated incidence of psychosis. Incidence estimates varied between 0.16 and 10.05% between included samples, which can be partially attributed to notable differences in age, duration of follow-up, and outcome definition and method for case ascertainment. For instance, the average age at the outcome for two studies was in the mid-twenties^31,34^, whereas for two other studies, it was in the mid-sixties to late seventies^28,30^. Furthermore, follow-up durations in these studies ranged from 7.3 to 24.9 years, with Laukkanen et al. having the longest follow-up and showing the highest incidence of psychosis, and Wium-Andersen et al. having the shortest follow-up and showing the lowest incidence. With regard to the outcome assessments, the cohorts used by Wium-Andersen et al., with the lowest incidence, likely overlooked some community diagnoses as these can be made in private practice in Denmark, and could therefore be missed in national registries. However, these substantial overall differences in incidence did not translate into statistically significant heterogeneity in ORs for the main outcome, therefore suggesting that inflammation might have played a similar role in all included cohorts.

Finally, however similarly to most previous research in immunopsychiatry, the included studies only measured CRP at baseline once, so this one measure may not accurately reflect long-term variability in CRP levels.

In conclusion, this meta-analysis of four prospective, population-based studies provides some evidence for a longitudinal association between high CRP levels (>3 mg/L) at baseline and greater risk for developing psychosis at follow-up. There was some indication that the effect sizes were larger for younger compared with older samples. However, these analyses included a limited number of cases of psychosis, and the results using CRP as continuous or categorical variables were largely null. In future, further studies with a larger number of cases are required before definitive conclusions can be drawn about the longitudinal association between CRP and psychosis risk.

## Methods

### Systematic literature search

We conducted a systematic review in line with the Preferred Reporting Items for Systematic Reviews and Meta-Analyses (PRISMA) guidelines^58^. Two separate databases, PubMed and Google Scholar, were searched from their inception to the 1^st^ of September 2020 for published, prospective studies of CRP levels and schizophrenia or related psychoses using the following search terms (* denotes wildcard terms):

(longitudinal OR cohort OR prospective*) AND (CRP OR “C-reactive protein” OR hs-CRP) AND (schizophreni* OR psychosis OR psychotic).

Google Scholar was used as a source of grey literature^59^. As suggested in ref. ^59^, the first 200 search results from Google Scholar were screened. The search was limited to studies based on human participants. The electronic search was complemented by hand-searching of meta-analyses and review articles and by contacting study authors. Abstracts were screened, and full texts of relevant studies were retrieved.

### Selection criteria

The inclusion criteria were:

- Longitudinal study design with data on CRP at baseline and psychosis at follow-up.
- CRP levels measured in plasma or serum at baseline using a standard laboratory method.
- Psychosis or schizophrenia assessed at follow-up using a standardized methodology; e.g., interview-assessed, or clinical diagnosis from electronic health records.
- Population-based samples, e.g., cohort or community-based, representative of the general population.

The exclusion criteria were:

- Cross-sectional studies or longitudinal studies based on particular groups of individuals selected on the basis of, e.g., a specific demographic characteristic, diagnosis of a certain disease/disorder, or a specific infection.
- Non-human subjects.
- Studies exclusively based in settings other than community (e.g., hospitalized).

When multiple articles were identified from a single cohort, the article with the largest sample or longest follow-up was included. Two authors (LB and EFO) independently applied the inclusion/exclusion criteria and selected the final studies for this review.

### Data extraction including collection of unpublished results

For each study, we extracted information on the OR for psychosis at follow-up, sample size, number of incident cases of psychosis at follow-up, length of follow-up, cohort, method of case ascertainment, country, age at baseline (CRP sampling), age at follow-up (psychosis diagnosis), sex, ethnicity, and definition of outcome.

We contacted all corresponding authors of the included studies and requested unpublished results. Specifically, for each study, we obtained the ORs for psychosis at follow-up associated with baseline CRP levels, coding CRP as:

- a binary variable (>3 mg/L versus ≤3 mg/L);
- a continuous variable, after log-transformation to correct right skew (per standard deviation [SD] increase);
- a categorical variable (≤1 mg/L [reference]; >1 and ≤3 mg/L; >3 mg/L.

ORs were obtained both before and after adjustments for potential confounding by age, sex, BMI; and after further adjustment for smoking, alcohol consumption, and SES, if available. These were selected as potential confounders as they are associated with psychosis and inflammation (please see discussion). Authors were also asked to provide unadjusted and adjusted ORs after excluding subjects with potential infection at baseline (defined as CRP > 10 mg/L). In addition, in the study by Wium-Andersen et al.^28^ ORs were adjusted for type of CRP assay, as two different methods were used for analysing CRP levels. Wium-Andersen et al. used education and income levels as measures for SES, while Metcalf et al. used maternal education levels as a proxy for SES. Perry et al. had no alcohol and smoking data available at baseline at the time of exposure measurement. However, since the exposure was measured at age 9 y, it is unlikely that many participants would have smoked or regularly consumed alcohol in this sample.

The methodological quality of each included study was assessed using the Newcastle-Ottawa Scale (NOS) for cohort studies^36,60^.

### Data synthesis and statistical analysis

Random effects models were used to meta-analyse ORs in order to address differences due to methodology and sample size. ORs were used as the preferred statistic over others (e.g., hazard ratios), due to uncertainties around the exact time of outcome in some samples. For instance, ALSPAC cohort participants^34^ were assessed for psychosis at age 24, but it is unclear when the outcome had first occurred. Register-based studies often relied on hospitalization primarily to identify cases^28,30,31^. While hospitalization is a useful proxy for illness time of onset, it may still not accurately reflect exact time of onset as not all patients are hospitalized and often there is a considerable delay between illness manifestation and help-seeking from healthcare professionals.

Random effects models were fitted using the restricted maximum-likelihood estimator, which has been shown to be effective in small meta-analytic samples where sample sizes are significantly diverse^61^. ORs were log-transformed for meta-analysis and then back-transformed for plotting in forest plots. Meta-analyses of ORs were carried out using the *metafor* package^62^ in R^63^.

Heterogeneity between studies was measured using the *I*^2^ statistic, which reflects the percentage of the variability in effect estimates that is due to heterogeneity. Heterogeneity was tested using Cochran’s *Q* Test^64^. *p* < 0.05 two tailed was considered statistically significant. Publication bias could not be assessed due to the small number of included studies.

## Supplementary information

Supplementary Information

## Data Availability

The dataset for this paper is freely available on Code Ocean, together with the R analysis codes, so that the analyses can be reproduced on the go at https://codeocean.com/capsule/3849634/tree.

## Code availability

The dataset for this paper is freely available on Code Ocean, together with the R analysis codes, so that the analyses can be reproduced on the go at https://codeocean.com/capsule/3849634/tree.

## References

[1] Dalman C (2008). Infections in the CNS during childhood and the risk of subsequent psychotic illness: a cohort study of more than one million Swedish subjects. Am. J. Psychiatry.

[2] Leask SJ, Done DJ, Crow TJ (2002). Adult psychosis, common childhood infections and neurological soft signs in a national birth cohort. Br. J. Psychiatry.

[3] Koponen H (2004). Childhood central nervous system infections and risk for schizophrenia. Eur. Arch. Psychiatry Clin. Neurosci..

[4] Khandaker GM, Zimbron J, Dalman C, Lewis G, Jones PB (2012). Childhood infection and adult schizophrenia: a meta-analysis of population-based studies. Schizophrenia Res..

[5] Sørensen HJ, Mortensen EL, Reinisch JM, Mednick SA (2009). Association between prenatal exposure to bacterial infection and risk of schizophrenia. Schizophrenia Bull..

[6] Brown AS (2000). Maternal exposure to respiratory infections and adult schizophrenia spectrum disorders: a prospective birth cohort study. Schizophrenia Bull..

[7] Babulas V, Factor-Litvak P, Goetz R, Schaefer CA, Brown AS (2006). Prenatal exposure to maternal genital and reproductive infections and adult schizophrenia. Am. J. Psychiatry.

[8] Nielsen PR, Laursen TM, Mortensen PB (2013). Association between parental hospital-treated infection and the risk of schizophrenia in adolescence and early adulthood. Schizophrenia Bull..

[9] Potvin S (2008). Inflammatory cytokine alterations in schizophrenia: a systematic quantitative review. Biol. Psychiatry.

[10] Miller BJ, Buckley P, Seabolt W, Mellor A, Kirkpatrick B (2011). Meta-analysis of cytokine alterations in schizophrenia: clinical status and antipsychotic effects. Biol. Psychiatry.

[11] Miller BJ, Gassama B, Sebastian D, Buckley P, Mellor A (2013). Meta-analysis of lymphocytes in schizophrenia: clinical status and antipsychotic effects. Biol. Psychiatry.

[12] Goldsmith DR, Rapaport MH, Miller BJ (2016). A meta-analysis of blood cytokine network alterations in psychiatric patients: comparisons between schizophrenia, bipolar disorder and depression. Mol. Psychiatry.

[13] Miller BJ, Culpepper N, Rapaport MH (2014). C-reactive protein levels in schizophrenia: a review and meta-analysis. Clin. Schizophr. Relat. Psychoses.

[14] Fernandes B (2016). C-reactive protein is increased in schizophrenia but is not altered by antipsychotics: meta-analysis and implications. Mol. Psychiatry.

[15] Pillinger T (2019). A Meta-analysis of immune parameters, variability, and assessment of modal distribution in psychosis and test of the immune subgroup hypothesis. Schizophrenia Bull..

[16] Upthegrove R, Manzanares-Teson N, Barnes NM (2014). Cytokine function in medication-naive first episode psychosis: a systematic review and meta-analysis. Schizophrenia Res..

[17] Danesh J (2000). Low grade inflammation and coronary heart disease: prospective study and updated meta-analyses. Bmj.

[18] Visser M, Bouter LM, McQuillan GM, Wener MH, Harris TB (1999). Elevated C-reactive protein levels in overweight and obese adults. JAMA.

[19] von Känel R (2007). Evidence for low-grade systemic proinflammatory activity in patients with posttraumatic stress disorder. J. Psychiatr. Res..

[20] Osimo EF, Baxter LJ, Lewis G, Jones PB, Khandaker GM (2019). Prevalence of Low-grade Inflammation in Depression: a systematic review and meta-analysis of CRP levels. Psychological Med..

[21] Osimo EF, Cardinal RN, Jones PB, Khandaker GM (2018). Prevalence and correlates of low-grade systemic inflammation in adult psychiatric inpatients: an electronic health record-based study. Psychoneuroendocrinology.

[22] Wysokiński A, Margulska A, Strzelecki D, Kłoszewska I (2015). Levels of C-reactive protein (CRP) in patients with schizophrenia, unipolar depression and bipolar disorder. Nord. J. Psychiatry.

[23] De Berardis D (2006). The role of C-reactive protein in mood disorders. Int J. Immunopathol. Pharm..

[24] De Berardis D (2017). Alexithymia, suicide ideation, C-reactive protein, and serum lipid levels among outpatients with generalized anxiety disorder. Arch. Suicide Res..

[25] De Berardis D (2008). Evaluation of C-reactive protein and total serum cholesterol in adult patients with bipolar disorder. Int J. Immunopathol. Pharm..

[26] Orsolini L (2018). Protein-C reactive as biomarker predictor of schizophrenia phases of illness? A systematic review. Curr. Neuropharmacol..

[27] Menard, S. *Handbook of lOngitudinal Research: Design, Measurement, and Analysis*. (Elsevier, 2007).

[28] Wium-Andersen MK, Ørsted DD, Nordestgaard BG (2014). Elevated C-reactive protein associated with late-and very-late-onset schizophrenia in the general population: a prospective study. Schizophrenia Bull..

[29] Khandaker GM, Pearson RM, Zammit S, Lewis G, Jones PB (2014). Association of serum interleukin 6 and C-reactive protein in childhood with depression and psychosis in young adult life: a population-based longitudinal study. JAMA Psychiatry.

[30] Laukkanen T, Laukkanen JA, Kunutsor SK (2018). Sauna bathing and risk of psychotic disorders: a prospective cohort study. Med. Princ. Pract..

[31] Metcalf SA (2017). Serum C-reactive protein in adolescence and risk of schizophrenia in adulthood: a prospective birth cohort study. Brain Behav. Immun..

[32] Francesconi M (2020). Internalising symptoms mediate the longitudinal association between childhood inflammation and psychotic-like experiences in adulthood. Schizophrenia Res..

[33] Miller, B. J. et al. Inflammation, hippocampal volume, and cognition in schizophrenia: results from the Northern Finland Birth Cohort 1966. *Eur. Arch. Psychiatry Clin. Neurosci*. 10.1007/s00406-020-01134-x (2020). 10.1007/s00406-020-01134-x 32382794

[34] Perry BI, Zammit S, Jones PB, Khandaker GM (2021). Childhood inflammatory markers and risks for psychosis and depression at age 24: examination of temporality and specificity of association in a population-based prospective birth cohort. Schizophrenia Res..

[35] Pearson TA (2003). Markers of inflammation and cardiovascular disease: application to clinical and public health practice: a statement for healthcare professionals from the Centers for Disease Control and Prevention and the American Heart Association. Circulation.

[36] Stang A (2010). Critical evaluation of the Newcastle-Ottawa scale for the assessment of the quality of nonrandomized studies in meta-analyses. Eur. J. Epidemiol..

[37] Steiner J (2020). Innate immune cells and C-reactive protein in acute first-episode psychosis and schizophrenia: relationship to psychopathology and treatment. Schizophr. Bull..

[38] Inoshita M (2016). A significant causal association between C-reactive protein levels and schizophrenia. Sci. Rep..

[39] Lin BD (2019). Assessing causal links between metabolic traits, inflammation and schizophrenia: a univariable and multivariable, bidirectional Mendelian-randomization study. Int. J. Epidemiol..

[40] Hartwig FP, Borges MC, Horta BL, Bowden J, Davey Smith G (2017). Inflammatory Biomarkers and Risk of Schizophrenia: A 2-Sample Mendelian Randomization Study. JAMA Psychiatry.

[41] Astle, W. J. et al. The allelic landscape of human blood cell trait variation and links to common complex disease. *Cell* **167**, 1415–1429.e1419 (2016). 10.1016/j.cell.2016.10.042 PMC5300907 27863252

[42] Khandaker GM (2019). Commentary: Causal associations between inflammation, cardiometabolic markers and schizophrenia: the known unknowns. Int. J. Epidemiol..

[43] Perry, B. I. et al. Inflammatory network alterations in schizophrenia, depression and bipolar disorder: a bi-directional two-sample Mendelian randomization study. *Under review* (2021). 10.1016/j.bbi.2021.07.009 PMC7612947 34280516

[44] Cho M (2019). Adjunctive use of anti-inflammatory drugs for schizophrenia: a meta-analytic investigation of randomized controlled trials. Aust. N.Z. J. Psychiatry.

[45] Kessler RC (2007). Age of onset of mental disorders: a review of recent literature. Curr. Opin. Psychiatry.

[46] Wörns MA, Victor A, Galle PR, Höhler T (2006). Genetic and environmental contributions to plasma C-reactive protein and interleukin-6 levels – a study in twins. Genes Immun..

[47] Radua J (2018). What causes psychosis? An umbrella review of risk and protective factors. World Psychiatry.

[48] Aleman A, Kahn RS, Selten J-P (2003). Sex differences in the risk of schizophrenia: evidence from meta-analysis. Arch. Gen. Psychiatry.

[49] Greenfield JR (2004). Obesity is an important determinant of baseline serum C-reactive protein concentration in monozygotic twins, independent of genetic influences. Circulation.

[50] Timpson NJ (2011). C-reactive protein levels and body mass index: elucidating direction of causation through reciprocal Mendelian randomization. Int. J. Obes..

[51] Sormunen E (2018). F135. Body Mass Index Trajectories in Childhood and Risk for Non-Affective Psychosis – a General Population Cohort Study. Schizophrenia Bull..

[52] Bulik-Sullivan B (2015). An atlas of genetic correlations across human diseases and traits. Nat. Genet..

[53] Nettis MA (2019). Metabolic-inflammatory status as predictor of clinical outcome at 1-year follow-up in patients with first episode psychosis. Psychoneuroendocrinology.

[54] Osimo EF (2021). Inflammatory and cardiometabolic markers at presentation with first episode psychosis and long-term clinical outcomes: a longitudinal study using electronic health records. Brain Behav. Immun..

[55] Compton MT (2009). Association of pre-onset cannabis, alcohol, and tobacco use with age at onset of prodrome and age at onset of psychosis in first-episode patients. Am. J. Psychiatry.

[56] Cochrane Collaboration. *10*.*4. 3.1 Recommendations on testing for funnel plot asymmetry*, https://handbook-5-1.cochrane.org/chapter_10/10_4_3_1_recommendations_on_testing_for_funnel_plot_asymmetry.htm (2011).

[57] Wener MH, Daum PR, McQuillan GM (2000). The influence of age, sex, and race on the upper reference limit of serum C-reactive protein concentration. J. Rheumatol..

[58] Moher D, Liberati A, Tetzlaff J, Altman DG, Group P (2009). Preferred reporting items for systematic reviews and meta-analyses: the PRISMA statement. PLoS Med..

[59] Haddaway NR, Collins AM, Coughlin D, Kirk S (2015). The role of Google scholar in evidence reviews and its applicability to grey literature searching. PLoS ONE.

[60] Higgins, J. P. T., Green, S. & Cochrane Collaboration. *Cochrane Handbook for Systematic Reviews of Interventions* (Wiley-Blackwell, 2008).

[61] Brannick MT, Yang L-Q, Cafri G (2011). Comparison of weights for meta-analysis of r and d under realistic conditions. Organ. Res. Methods.

[62] Viechtbauer W (2010). Conducting meta-analyses in R with the metafor package. J. Stat. Softw..

[63] R: A language and environment for statistical computing [Software] (R Foundation for Statistical Computing, Vienna, Austria, 2020).

[64] Higgins JP, Thompson SG (2002). Quantifying heterogeneity in a meta-analysis. Stat. Med..

[65] Sullivan SA (2020). A population-based cohort study examining the incidence and impact of psychotic experiences from childhood to adulthood, and prediction of psychotic disorder. Am. J. Psychiatry.

