# Supplementary material for: Longitudinal association between CRP levels and risk of psychosis: a meta-analysis of population-based cohort studies
Source: NPJ Schizophr. 2021 May 28;7:31. doi: 10.1038/s41537-021-00161-4 (PMC8163886; doi:10.1038/s41537-021-00161-4)
Supplement: Supplementary file 1 — Supplementary Information [file 41537_2021_161_MOESM1_ESM.pdf]

Longitudinal association between C-reactive protein levels and risk of psychosis: a systematic review and meta-analysis of population-based, prospective cohort studies

Emanuele F. Osimo et al, 2021

Table of contents:

Supplementary Tables 2

Supplementary Table 1: Quality assessment of included studies according to the Newcastle-Ottawa quality assessment scale for cohort studies 2

Supplementary Figures 3

Supplementary Figure 1: PRISMA diagram of literature search 3

Supplementary Figure 2: Odds ratios for psychosis at follow-up for individuals with high (>3 mg/L) and medium (1-3 mg/L), as compared to low (≤1 mg/L), CRP levels at baseline 4

Supplementary Figure 3: Odds ratios for psychosis at follow-up for individuals with high (>3 mg/L), as compared to low (≤3 mg/L), CRP levels at baseline – after excluding individuals with baseline CRP >10mg/L 5

Supplementary Figure 4: Odds ratios for psychosis at follow-up per SD increase in CRP levels at baseline – after excluding individuals with baseline CRP >10mg/L 6

Supplementary Figure 5: Odds ratios for psychosis at follow-up for individuals with high (>3 mg/L) and medium (1-3 mg/L), as compared to low (≤1 mg/L), CRP levels at baseline – after excluding individuals with baseline CRP >10mg/L 7

References: 8

Supplementary Tables

Supplementary Table 1: Quality assessment of included studies according to the Newcastle-Ottawa quality assessment scale for cohort studies

The Newcastle-Ottawa Scale (NOS) considers study quality based on three domains: 1) Quality of subject '**selection'** (representativeness of the exposed cohort, selection of the non-exposed cohort, ascertainment of exposure, demonstration that outcome of interest was not present at start of study, **maximum 4 points**); 2) Quality of '**comparability'** (study controls for most important factor and any additional factor, **maximum 2 points**); 3) Quality of '**outcome**' (including assessment of outcome, length of follow-up, adequacy of follow-up, **maximum 3 points**). A maximum of 9 points was therefore considered.

Thresholds for converting NOS rating to Agency for Healthcare Research and Quality standards (good, fair, poor) (Pillinger et al., 2019). *Good – 3 or 4 stars in Selection AND 1 or 2 in Comparability AND 2 or 3 in Outcome. Fair – 2 stars in Selection AND 1 or 2 in Comparability AND 2 or 3 in Outcome. Poor – 0 or 1 stars in Selection OR 0 in Comparability OR 0 or 1 in Outcome.*

	**Selection**				**Comparability**	**Outcome**			**HRQ score**
	**Representative-ness**	**Selection**	**Exposure**	**Outcome not at start**	**Comparable cohorts**	**Assessment**	**Follow-up length of time**	**Loss to follow-up**	
**Wium-Andersen et al, 2014**	1	1	1	1	2	1	1	1	good
**Metcalf et al, 2017**	1	1	1	0	2	1	1	1	good
**Laukkanen et al, 2018**	1	1	1	1	2	1	1	1	good
**Perry et al, 2021**	1	1	1	0	2	1	1	0	good

Supplementary Figures

Supplementary Figure 1: PRISMA diagram of literature search

PRISMA 2009 Flow Diagram

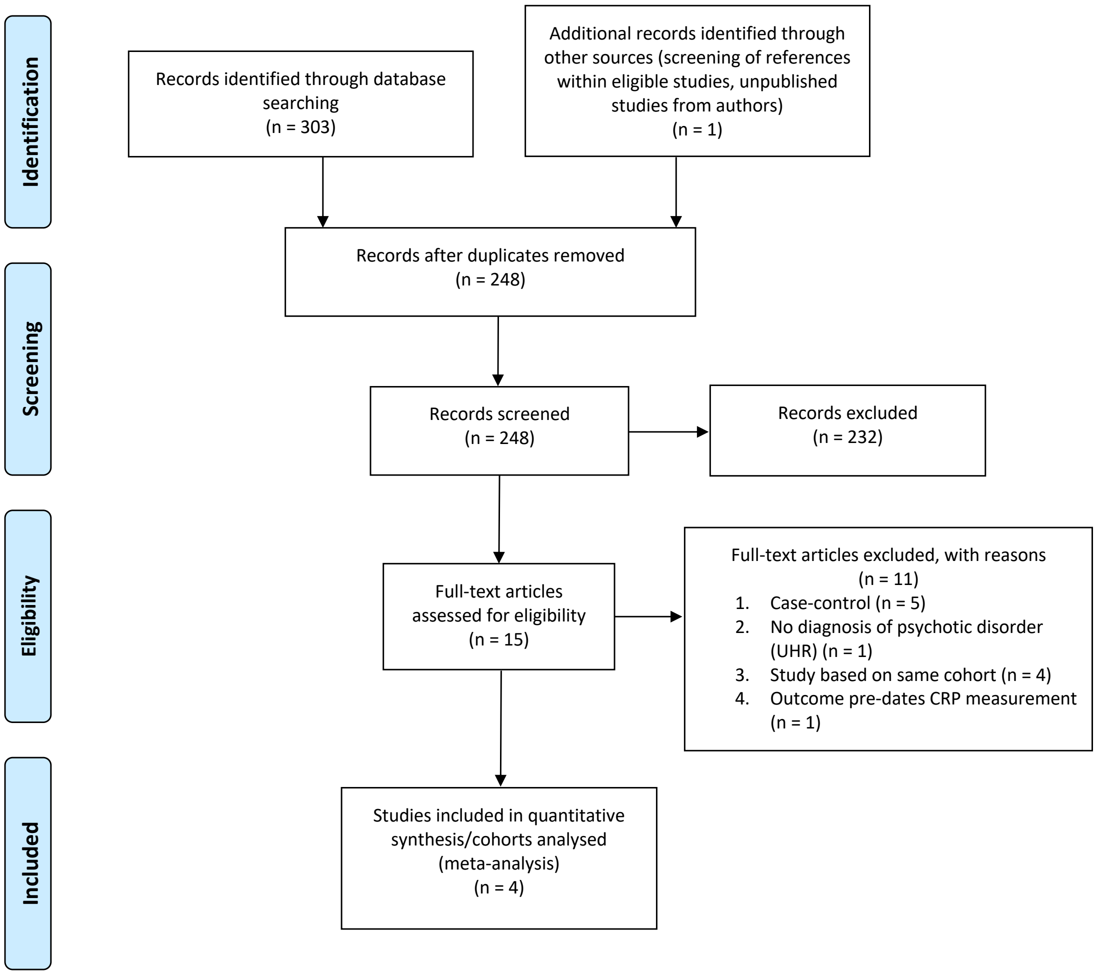

From: Moher D, Liberati A, Tetzlaff J, Altman DG, The PRISMA Group (2009). *P*referred *R*eporting *I*tems for *S*ystematic Reviews and *M*eta-*A*nalyses: The PRISMA Statement. PLoS Med 6(7): e1000097. doi:10.1371/journal.pmed1000097

For more information, visit www.prisma-statement.org.

CRP, C-reactive protein; UHR, ultra-high risk

Supplementary Figure 2: Odds ratios for psychosis at follow-up for individuals with high (>3 mg/L) and medium (1-3 mg/L), as compared to low (<1 mg/L), CRP levels at baseline

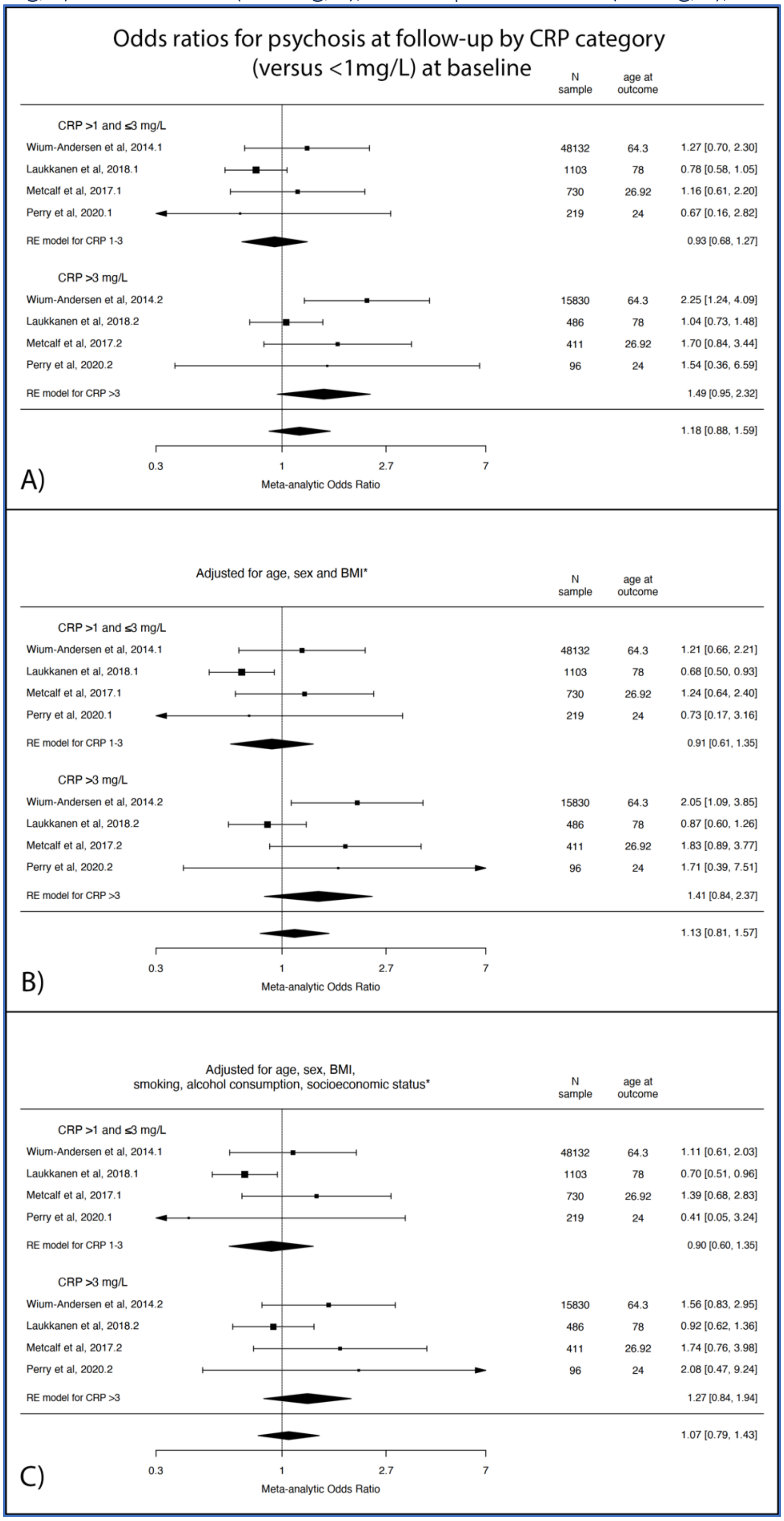

BMI, body mass index; CRP, C-reactive protein; mg/L, milligrams per litre

Supplementary Figure 3: Odds ratios for psychosis at follow-up for individuals with high (>3 mg/L), as compared to low (≤3 mg/L), CRP levels at baseline – after excluding individuals with baseline CRP >10mg/L

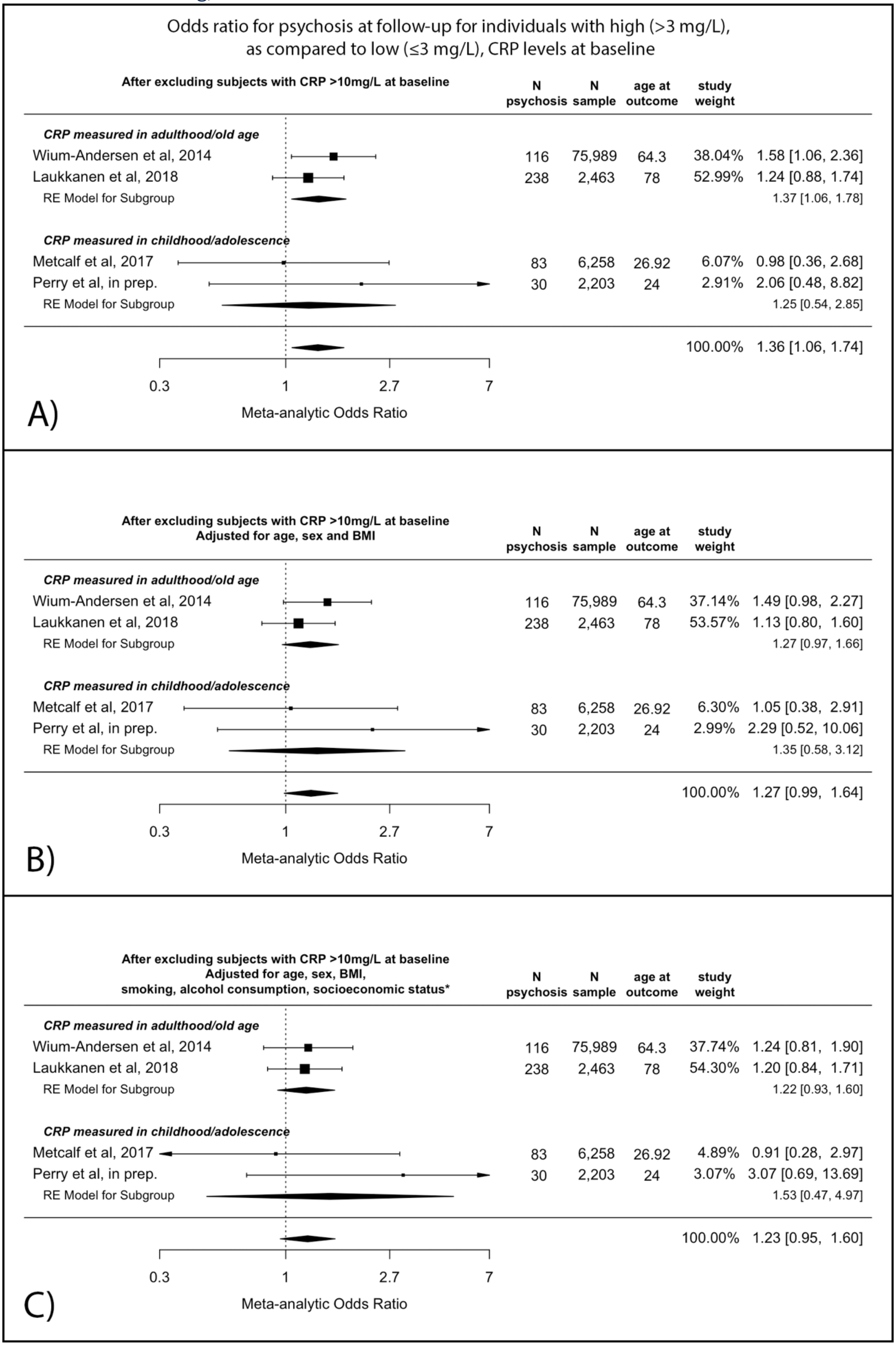

BMI, body mass index; CRP, C-reactive protein; mg/L, milligrams per litre

Supplementary Figure 4: Odds ratios for psychosis at follow-up per SD increase in CRP levels at baseline – after excluding individuals with baseline CRP >10mg/L

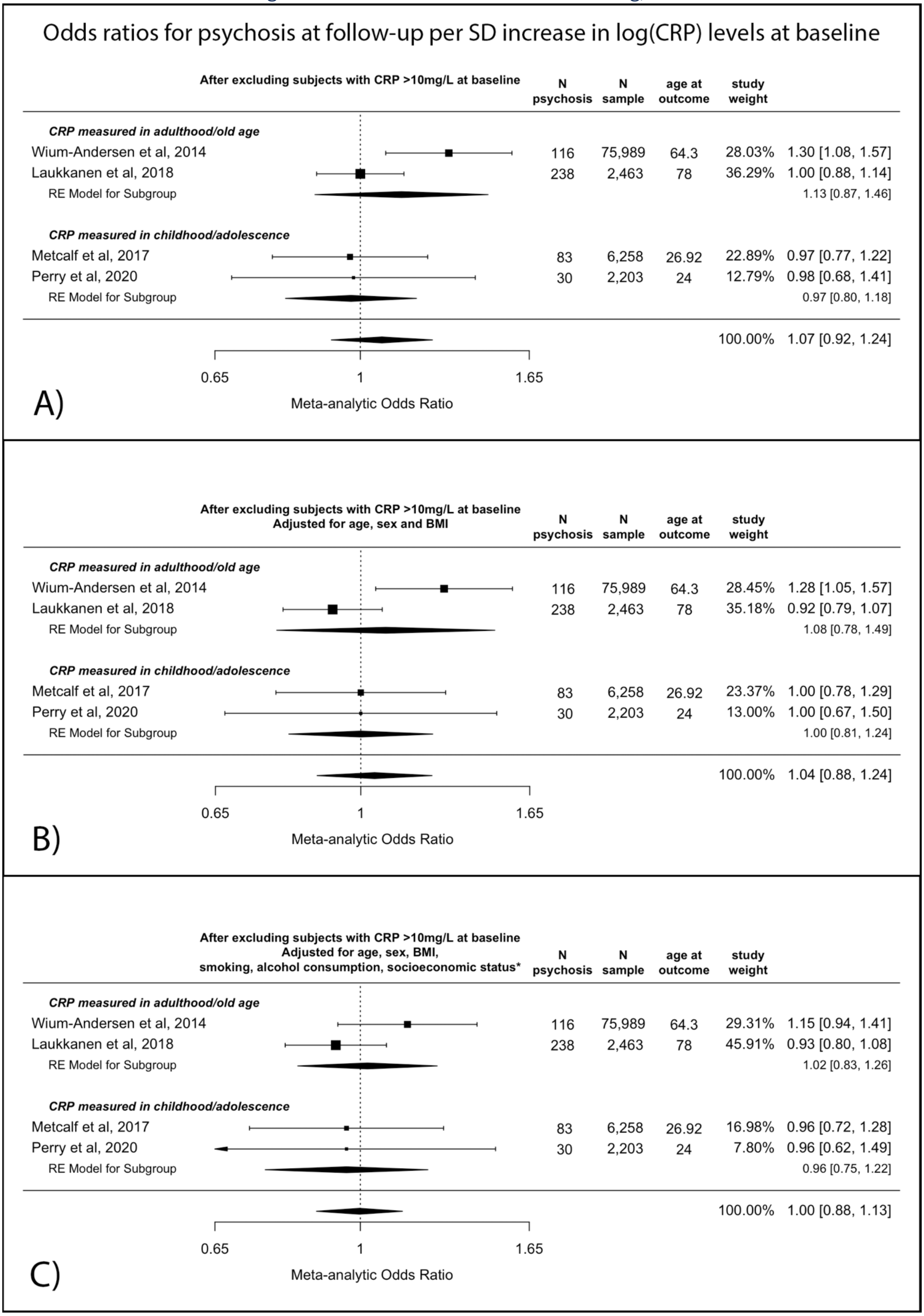

BMI, body mass index; CRP, C-reactive protein; mg/L, milligrams per litre

Supplementary Figure 5: Odds ratios for psychosis at follow-up for individuals with high (>3 mg/L) and medium (1-3 mg/L), as compared to low (<1 mg/L), CRP levels at baseline – after excluding individuals with baseline CRP >10mg/L

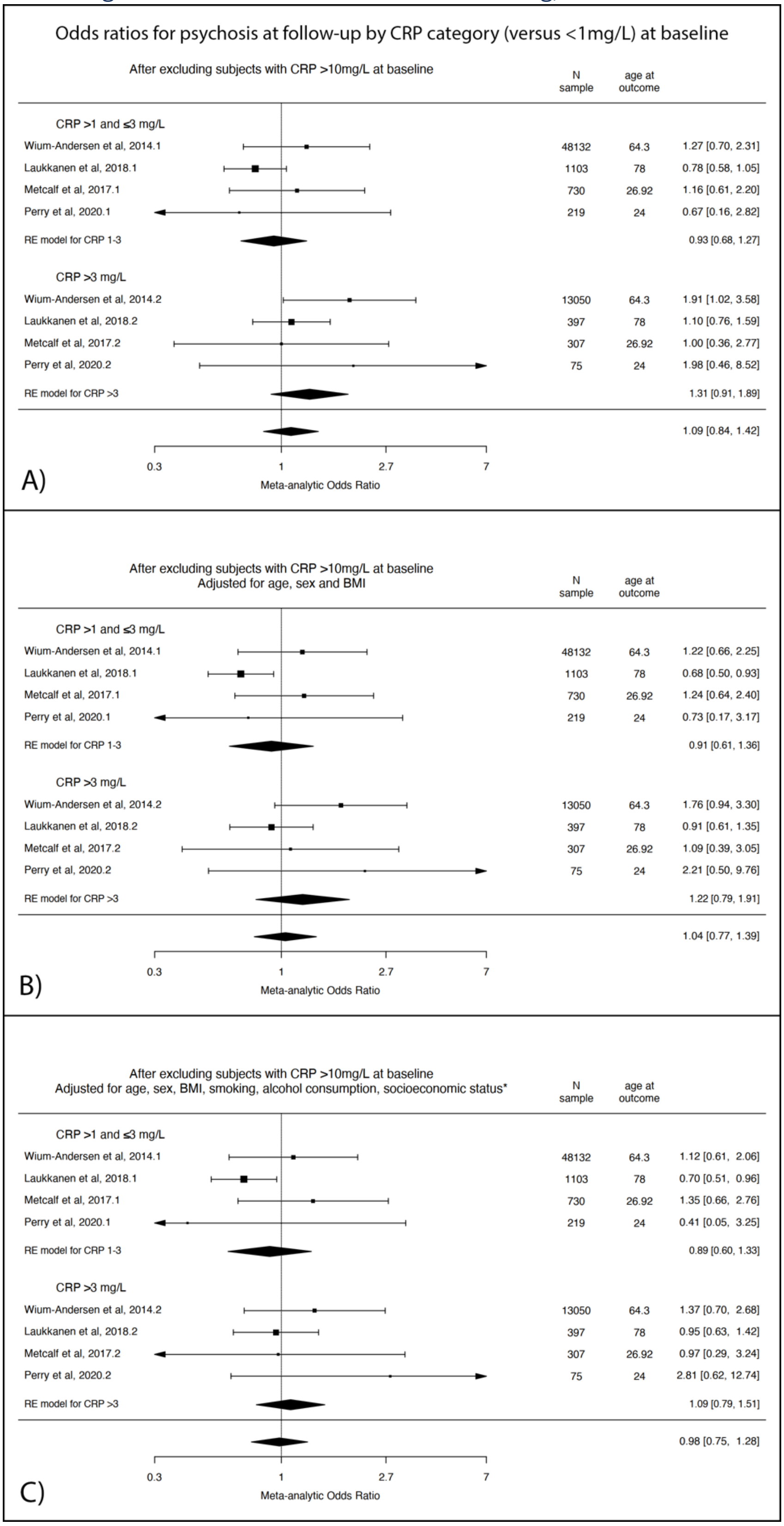

BMI, body mass index; CRP, C-reactive protein; mg/L, milligrams per litre

References:

Pillinger, T., Osimo, E.F., Brugger, S., Mondelli, V., McCutcheon, R.A., Howes, O.D., 2019. A Meta-analysis of Immune Parameters, Variability, and Assessment of Modal Distribution in Psychosis and Test of the Immune Subgroup Hypothesis. Schizophrenia Bulletin 45, 1120–1133.
